# Supplementary material for: Molecular rationale for antibody-mediated targeting of the hantavirus fusion glycoprotein
Source: eLife. 2020 Dec 22;9:e58242. doi: 10.7554/eLife.58242 (PMC7755396; doi:10.7554/eLife.58242)
Supplement: Supplementary file 1. — Table S2. Cryo-EM tomography data collection, sub-tomogram reconstruction, and fitting statistics. [file elife-58242-supp1.docx]

Table S1. Crystallographic data collection and refinement statistics for Fab P-4G2−PUUV Gc

|  | Fab P-4G2**−**PUUV Gc |
| --- | --- |
| **Data collection** |  |
| Space group | *P* 1 21 1 |
| Cell dimensions |  |
| *a*, *b*, *c* (Å) | 97.2, 50.5, 124.5 |
| *a*, *b*, *g*  (°) | 90.0, 107.7, 90.0 |
| Resolution (Å) | 92.6-3.50 (3.63-3.50)* |
| *R*_merge_ | 0.413 (-) |
| *R*_pim_ | 0.140 (0.426) |
| I/σ I | 4.6 (1.9) |
| CC_1/2_ | 0.96 (0.65) |
| Completeness (%) | 99.7 (97.5) |
| Multiplicity | 9.6 (9.0) |
| **Refinement** |  |
| Resolution (Å) | 92.63-3.50 (3.77-3.50) |
| No. reflections | 14,903 |
| *R*_work_ / *R*_free_ | 0.217 / 0.264 |
| No. atoms |  |
| Protein | 6,464 |
| Ligand/ion | 32 |
| Water | 0 |
| *B*-factors |  |
| Protein | 70.6 |
| Ligand/ion | 58.2 |
| Ramachandran plot (%) |  |
| Favored region | 94.98 |
| Allowed region | 5.02 |
| Outliers | 0 |
| R.m.s deviations |  |
| Bond lengths (Å) | 0.001 |
| Bond angles (°) | 0.454 |

* Highest resolution shell is shown in parentheses

**Table S2.** Cryo-EM tomography data collection, sub-tomogram reconstruction, and fitting statistics

|  | **PUUV VLP** | **PUUV VLP +P-4G2 fab** | |  |
| --- | --- | --- | --- | --- |
| Tilt range (degrees) | -30–60 | -30–60 | |  |
| Tilt increment (degrees) | 3 | 3 | |  |
| Frames per tilt | 6 | 6 | |  |
| Electron exposure (e⁻/ Å²/tilt) | 4.5 | 4.5 | |  |
| Total electron exposure (e⁻/ Å²) | 140 | 140 | |  |
| Pixel size (Å) | 1.77 | 1.76 | |  |
| Defocus range (μm) | 2.8–4.0 | 2.8–4.0 | |  |
| Tilt series | 33 | 59 | |  |
| Tomograms | 33 | 59 | |  |
| VLP sub-volumes | 42 | 79 | |  |
| Reconstruction | GP spike | GP spike | GP spike+P-4G2 | |
| EMDB ID | EMD- xxxx | EMD- xxxx | EMD- xxxx | |
| Spike sub-volumes | 4,323 | 1,976 | 1,721 | |
| Symmetry | C4 | C4 | C4 | |
| Pixel size (Å) | 3.52 | 3.52 | 3.52 | |
| Resolution (Å) | 13.9 | 14.3 | 13.4 | |
| Model-to-Map CC | 0.89^ | nd. | 0.91* | |

CC: local map-to-map cross-correlation from Chimera fitmap

* Fitted structure: Fab P-4G2–PUUV Gc complex crystal structure

^ Fitted structure: PUUV Gn (from PDB 5FXU)+PUUV Gc_Gc_–_P-4G2_

nd.: Not determined
