# Supplementary material for: Molecular rationale for antibody-mediated targeting of the hantavirus fusion glycoprotein
Source: eLife. 2020 Dec 22;9:e58242. doi: 10.7554/eLife.58242 (PMC7755396; doi:10.7554/eLife.58242)
Supplement: Transparent reporting form [file elife-58242-transrepform.docx]

***eLife’s* transparent reporting form**

We encourage authors to provide detailed information *within their submission* to facilitate the interpretation and replication of experiments. Authors can upload supporting documentation to indicate the use of appropriate reporting guidelines for health-related research (see [EQUATOR Network](http://www.equator-network.org/%20)), life science research (see the [BioSharing Information Resource](https://biosharing.org/)), or the [ARRIVE guidelines](http://www.plosbiology.org/article/info:doi/10.1371/journal.pbio.1000412) for reporting work involving animal research. Where applicable, authors should refer to any relevant reporting standards documents in this form.

If you have any questions, please consult our Journal Policies and/or contact us: [editorial@elifesciences.org](mailto:editorial@elifesciences.org).

**Sample-size estimation**

- You should state whether an appropriate sample size was computed when the study was being designed
- You should state the statistical method of sample size computation and any required assumptions
- If no explicit power analysis was used, you should describe how you decided what sample (replicate) size (number) to use

This paper reports an X-ray crystal structure and two cryoEM reconstructions. Crystallography and electron microscopy data processing and refinement statistics can be found in Supplementary File 1. Further details regarding data processing can be found in the materials and methods, sections ‘Crystallization and structure determination’, ‘Cryo-EM grid preparation, data acquisition and data processing’, and ‘Quantification of the frequency of lattice formation on VLP surface in the presence and absence of Fab P-4G2’. For the supporting functional assay, comprising the neutralization data presented in Figures 2 and 3, the details of the data analysis can be found in the legends of Figure 2 and Figure 3, and in materials and methods section ‘Hantavirus-pseudotyped VSV neutralization assay’.

Please outline where this information can be found within the submission (e.g., sections or figure legends), or explain why this information doesn’t apply to your submission:

**Replicates**

- You should report how often each experiment was performed
- You should include a definition of biological versus technical replication
- The data obtained should be provided and sufficient information should be provided to indicate the number of independent biological and/or technical replicates
- If you encountered any outliers, you should describe how these were handled
- Criteria for exclusion/inclusion of data should be clearly stated
- High-throughput sequence data should be uploaded before submission, with a private link for reviewers provided (these are available from both GEO and ArrayExpress)

Replicates are not applicable to the crystallography and electron microscopy data that comprise the primary results of this paper. For the supporting functional assay, comprising the neutralization data presented in Figures 2 and 3, each neutralization assay was carried out three times in duplicate. The details of the data analysis can be found in the legends of Figure 2 and Figure 3, and in materials and methods section ‘Hantavirus-pseudotyped VSV neutralization assay’.

Please outline where this information can be found within the submission (e.g., sections or figure legends), or explain why this information doesn’t apply to your submission:

**Statistical reporting**

- Statistical analysis methods should be described and justified
- Raw data should be presented in figures whenever informative to do so (typically when N per group is less than 10)
- For each experiment, you should identify the statistical tests used, exact values of N, definitions of center, methods of multiple test correction, and dispersion and precision measures (e.g., mean, median, SD, SEM, confidence intervals; and, for the major substantive results, a measure of effect size (e.g., Pearson's r, Cohen's d)
- Report exact p-values wherever possible alongside the summary statistics and 95% confidence intervals. These should be reported for all key questions and not only when the p-value is less than 0.05.

Crystallography and electron microscopy data processing and refinement statistics can be found in Supplementary File 1. Further details regarding data processing and analysis can be found in the materials and methods, sections ‘Crystallization and structure determination’, ‘Cryo-EM grid preparation, data acquisition and data processing’, and ‘Quantification of the frequency of lattice formation on VLP surface in the presence and absence of Fab P-4G2’.

Please outline where this information can be found within the submission (e.g., sections or figure legends), or explain why this information doesn’t apply to your submission:

(For large datasets, or papers with a very large number of statistical tests, you may upload a single table file with tests, Ns, etc., with reference to sections in the manuscript.)

**Group allocation**

- Indicate how samples were allocated into experimental groups (in the case of clinical studies, please specify allocation to treatment method); if randomization was used, please also state if restricted randomization was applied
- Indicate if masking was used during group allocation, data collection and/or data analysis

This paper reports an X-ray crystal structure and two cryoEM reconstructions. Crystallography and electron microscopy data processing and refinement statistics can be found in Supplementary File 1. Further details regarding data processing can be found in the materials and methods, sections ‘Crystallization and structure determination’, ‘Cryo-EM grid preparation, data acquisition and data processing’, and ‘Quantification of the frequency of lattice formation on VLP surface in the presence and absence of Fab P-4G2’. For the supporting functional assay, comprising the neutralization data presented in Figures 2 and 3, the details of the data analysis can be found in the legends of Figure 2 and Figure 3, and in materials and methods section ‘Hantavirus-pseudotyped VSV neutralization assay’.

Please outline where this information can be found within the submission (e.g., sections or figure legends), or explain why this information doesn’t apply to your submission:

**Additional data files (“source data”)**

- We encourage you to upload relevant additional data files, such as numerical data that are represented as a graph in a figure, or as a summary table
- Where provided, these should be in the most useful format, and they can be uploaded as “Source data” files linked to a main figure or table
- Include model definition files including the full list of parameters used
- Include code used for data analysis (e.g., R, MatLab)
- Avoid stating that data files are “available upon request”

Please indicate the figures or tables for which source data files have been provided:

Atomic coordinates and structure factors of the PUUV Gc-Fab P-4G2 complex crystal structure have been deposited in the PDB under accession code 6Z06. Cryo-EM reconstructions of the PUUV VLP surface alone and in the presence of Fab P-4G2 from areas of continuous and discontinuous lattice have been deposited in the EMDB at the EBI under accession codes **EMD-11966, EMD-11965 and EMD-11964,** respectively. Coordinates of protein structures fitted into these cryo-EM reconstructions have been deposited in the **PDB database** (accession code **7B09** for the Fab P-4G2-PUUV Gc and PUUV Gn fitted into **EMD-11964**, and accession code **7B0A** for PUUV Gc and PUUV Gn fitted into **EMD-11966**, respectively). All the deposited data will be released upon publication of the article.
